# Resolution of thrombocytopenia, but not polycythemia after ruxolitinib for polycythemia vera with detectable mutation in the exon 12 of the JAK2 gene

**DOI:** 10.1007/s12032-017-0891-8

**Published:** 2017-01-24

**Authors:** Grzegorz Helbig, Ryszard Wichary, Karolina Torba, Sławomira Kyrcz-Krzemień

**Affiliations:** Department of Hematology and Bone Marrow Transplantation, School of Medicine in Katowice, Medical University of Silesia, Dąbrowski Street 25, 40-032 Katowice, Poland

To the Editor

Data on safety and efficacy of ruxolitinib treatment in myeloproliferative neoplasms (MPN) with concomitant thrombocytopenia are scarce and limited to patients with myelofibrosis (MF). It was demonstrated that 11–16% of MF patients had platelet (PLT) count <50 × 10^9^/l at diagnosis and the rate of thrombocytopenia increases with disease progression. Thrombocytopenia in MF may be due to ineffective megakaryocytopoiesis as a result of bone marrow fibrosis or results from platelet sequestration and destruction in enlarged spleen [1]. Immune dysregulation with the production of immune complexes or antiplatelet antibodies may account for low platelet count in single cases [2]. It is noteworthy that thrombocytopenia remains an exceptional finding in polycythemia vera (PV).

Ruxolitinib is the first and only approved JAK inhibitor for the treatment of intermediate- and high-risk MF as well as for PV patients with inadequate response to or are intolerant of hydroxyurea. It should be mentioned that thrombocytopenia was identified as the dose-limiting toxicity for ruxolitinib in MF and was the most common cause of dose modifications. Grade 3/4 thrombocytopenia was rarely seen in PV patients receiving ruxolitinib [3].

There has been a limited experience with ruxolitinib in thrombocytopenic patients with MPN, and all available data were derived from studies in MF. Based on these preliminary results, the treatment with low dose of ruxolitinib (10 mg daily) was well tolerated and resulted in spleen reduction with PLT count improvement. To date, only few MF patients with PLT count of <50 × 10^9^/l were successfully treated with ruxolitinib [1, 4].

A 25-year-old female initiated a diagnostic workup due to progressive thrombocytopenia. Seven years earlier, she was diagnosed with polycythemia vera (PV) with detectable H538–K539delinsL mutation in the exon 12 of the JAK2 gene. Since her PV diagnosis she was phlebotomized every other month and receiving hydroxyurea and interferon with no effect. During the follow-up, she developed an isolated mild thrombocytopenia (110 × 10^9^/l). She did not present a prior history of immune thrombocytopenia or any other autoimmune disorders.

**Fig. 1:**
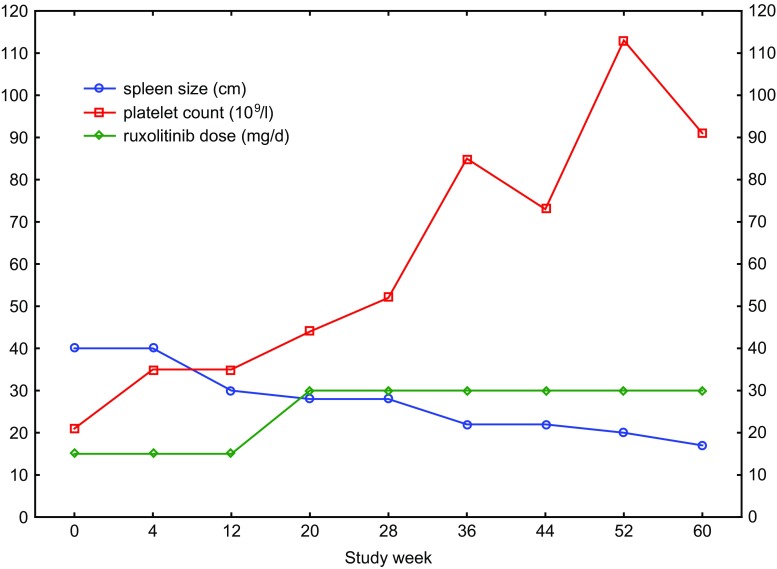
Graphical representation of platelet count, spleen size, and ruxolitinib daily dose during the disease course

On her current admission the white blood cell (WBC) count was 28.1 × 10^9^/l with the predominance of segmented cells in differential (78%). No myeloid precursors were found. Red blood cell (RBC) and PLT counts were 5.98 × 10^6^/µl and 21 × 10^9^/l, respectively. A massive splenomegaly (40 cm) was found on ultrasonography. On trephine biopsy her marrow cellularity was 100%. She had reticullin fibrosis of grade I and megakaryocytes were numerous with normal maturation. Blast cells were not seen. Cytogenetics detected normal diploid karyotype. She was found to have antiplatelet antibodies and received steroids with no PLT increase. Then, she was started with ruxolitinib at reduced dose of 15 mg daily due to grade 4 thrombocytopenia. A slow increase in PLT count was observed with concomitant spleen reduction. Five months later, her PLT count was 44 × 10^9^/l, and ruxolitinib dose was increased to 30 mg daily. At 15 months of ruxolitinib treatment, her PLT count was 93 × 10^9^/l with further spleen reduction (17 cm on ultrasound). Figure 1. However, her RBC and WBC counts were elevated, and she required regular phlebotomies. A repeated trephine biopsy remained unchanged, and the exon 12 mutation was still present.

To date, coexistence of PV with immune thrombocytopenic purpura (ITP) was reported in three cases. All patients were male >60 years old with the JAK2 V617F-positive PV and PLT counts ranging from 8 to 29 × 10^9^/l. Of note is that they did not exhibit a significant splenomegaly on physical examination, and ITP diagnosis was established by exclusion of other causes of thrombocytopenia. Moreover, low PLT count increased after steroids introduction [2]. Our PV patient presented with severe thrombocytopenia which was related to two independent mechanisms (1) the presence of autoimmunity and (2) hypersplenism. Of note is that steroids were ineffective. The incorporation of ruxolitinib at 15 mg daily resulted in slow platelet recovery without side effects. However no improvements of PV features were observed despite time-dependent dose increase and patient remains phlebotomy dependent. It seems likely that the increase in PLT count was most of all associated with reduction of spleen size. It was found that patients with immune thrombocytopenia have an increased Th17 population and defective function of regulatory T cells (Tregs) [5]. The recent data show that treatment with ruxolitinib resulted in a significant reduction of regulatory Tregs and polarization of CD4+ cells from a Th1 to a Th17 phenotype [6]. In this context, ruxolitinib treatment should rather deepen thrombocytopenia than improve it. These data may prove against the beneficial immunomodulatory effect of JAK inhibition in ITP.

To our best knowledge, this is the first report showing the recovery of severe thrombocytopenia after ruxolitinib for PV.
